# Introduction of structured physical examination skills to second year undergraduate medical students

**DOI:** 10.12688/f1000research.2-16.v1

**Published:** 2013-01-16

**Authors:** Rano M Piryani, P Ravi Shankar, Trilok P Thapa, Bal M Karki, Rishi K Kafle, Mahesh P Khakurel, Shital Bhandary

**Affiliations:** 1 KIST Medical College, Lalitpur, Kathmandu Valley, Nepal; 2 Patan Academy of Health Sciences, Lalitpur, Kathmandu Valley, Nepal

## Abstract

**Introduction:** Effective learning of physical examination skills (PES) requires suitable teaching and learning techniques and assessment methods. The Tribhuvan University (Nepal) curriculum recommends involving the departments of Medicine and Surgery in PES training (PEST) for second year students as a part of early clinical exposure. The project was developed to make teaching/learning of PES structured, involving eight clinical sciences departments and using appropriate methods for teaching and assessment in KIST Medical College, Nepal.

**Methods:** Irby’s three stages of clinical teaching model (Preparation, Teaching, Reflection), was applied for teaching. Skill acquisition was based on Millers’ learning pyramid at “show how level” and Dreyfus’ competency model at “competent level”. Teaching/learning was conducted in small groups. A tutorial, demonstration and practice (TDS) model was developed for teaching/learning techniques based on a simple five-step method for teaching clinical skills. Assessment of effectiveness of training was done at “reaction level” as per Kirkpatrick’s model based on students’ feedback, “shows how level” as per Miller’s pyramid of learning by OSCE and “competent level” as per Dreyfus’ model using retro-pre questionnaire.

**Results:** The analysis of retro-pre questionnaire based on the Dreyfus model found the average skill score (max score 184), before the introduction of the project module as 15.9 (median = 13.5) and after as 116.5 (median = 116). A paired t-test showed the difference to be statistically significant (100.5±23 and 95% CI 95.45 – 105.59). The average overall feedback score for the students on PES training based on seven items on a five point Likert scale was found to be 4.30. The mean total objective structured clinical examination (OSCE) score was 3.77 (SD+/- 0.33) out of 5; 80% of students scored more than 70%.

**Conclusion:** Students learned most of the skills with the implementation of the structured PES module and did well in the OSCE. Students and faculty were satisfied with the training and assessment.

## Introduction

Learning physical examination skills (PES) is an important aspect of undergraduate medical students’ training in the early clinical years ^1^. Effective clinical teaching and learning of PES requires appropriate teaching and learning techniques and assessment methods ^2^. KIST Medical College (KISTMC), Lalitpur, Nepal, is a newly established medical school in the private sector and admitted its first batch of students in November 2008. It follows the curriculum of Tribhuvan University Institute of Medicine (TU-IOM), Kathmandu, Nepal ^3^. The curriculum stresses early clinical exposure with the first year being devoted to the acquisition of history taking and communication skills and the second year to physical examination skills. The curriculum recommends involvement of the departments of Medicine and Surgery in PES training (PEST) for second year students as part of early clinical exposure (ECE). The methods for teaching/learning and assessment are not well defined in the curriculum.

The project was developed to make teaching-learning of PES structured, and involved eight clinical departments using appropriate teaching, learning and assessment methods. Students are provided with an opportunity to learn basic physical examination skills in gynecology and obstetrics, orthopedics, ear, nose and throat (ENT), ophthalmology and pediatrics, as well as general medicine and surgery in their second year Bachelor of Medicine and Bachelor of Surgery degrees (MBSS) so that they get sufficient time to learn reasoning, diagnostic, procedural, therapeutic and counseling skills during their clinical years (third, fourth and final year). The objective of the project was that at the end of the second year, students should be able to initiate and perform a basic physical examination of an adult suffering from medical, surgical, gynecology and obstetric, orthopedic, ENT and eye diseases, as well as a basic physical examination of a child.

## Methods

### I. Development of the module

Faculty members of departments involved in the project identified basic PES to be learnt by students. A checklist for each selected PES was prepared based on Hutichson’s Clinical Methods (22nd edition) ^4^ and was peer reviewed and finalized by a core project committee.

### II. Orientation of faculty members

All faculty members involved in teaching received teacher training before commencement of the module. They were oriented with regards to the implementation of the project in a mini-workshop. The details regarding grouping of students, the posting schedule of various groups in different departments in rotation, the approach to teaching and learning, the teaching-learning strategy and the assessment modalities were also shared with them.

### III. Approach to teaching

Physical examination skills involve psychomotor skills. For teaching physical examination skills, Irby’s three stages of clinical teaching were applied ^5^. These are: preparation (stage I), teaching (stage II) and reflection (stage III).

### IV. Approach to learning

Skills acquisition was based on Millers’ Learning Pyramid ^6^ at the “show how level” and Dreyfus’ competency model ^6^ at the “competent level”. Miller’s four levels of learning are:

1) Whether the learner has *knowledge* of the skill;

2) Whether the learner *knows how* the skill is performed;

3) Whether the learner *shows how* to perform the skill in a controlled or simulated setting; and

4) Finally, whether the learner actually *does* the skill in clinical practice.

The basic principle of the Dreyfus model is that the student progresses through five stages of proficiency in this specific order: novice, advanced beginner, competent, proficient, and expert ^6^.

### V. Teaching-learning strategies

Teaching-learning was conducted in small groups. The one hundred students were divided into seven groups of 14–15 students; each group was further divided into two subgroups of seven or eight students. Each group was posted for four weeks each in Medicine Units I and II, surgery, pediatrics, and gynecology and obstetrics, and each subgroup for two weeks in family medicine, ENT, ophthalmology and orthopedics in rotation. Students learned PES related to the cardiovascular system (CVS) and respiratory system (RSS) in Medicine Unit I, the peripheral nervous system (PNS) and central nervous system (CNS) in Medicine Unit II, the examination of the abdomen in Surgery, the musculoskeletal system (MS) in orthopedics, general PES in family medicine, obstetrics and gynecology examination in the obstetrics and gynecology department, ear, nose and throat examination in ENT and eye examination in ophthalmology. Structured PEST (S-PEST) sessions were held for four hours every Monday for 28 weeks between February and August 2011.

Based on the method used for teaching clinical skills in the American College of Surgeon’s advanced trauma life support course, a tutorial, demonstration and practice (TDP) model was developed. Each S-PEST session had three sub-sessions: Tutorial (T), Demonstration (D) and Practice (P). The ‘Tutorial’ element covered the overview by the faculty preceptor on skills to be taught; ‘Demonstration’ involved actually demonstrating each of the skills taught with a stepwise description, while ‘Practice’ involved performance/practice of each demonstrated skill by the students using a sequential description to be observed by the preceptor. This model follows five (conceptualization, visualization, verbalization, practice and correction and reinforcement) of the seven psychomotor teaching principles based on the taxonomy of psychomotor domain (the other two being skill mastery and skill autonomy) ^7^.

In most sessions, demonstration and performance/practice were conducted on real patients either in the ward or outpatient department (OPD). Some sessions were conducted on simulated patients, while in a few sessions the students themselves consented to be simulated patients.

### VI. Assessment

Assessment of PES training effectiveness was conducted at Kirkpatrick’s level 1 - Reaction (see below for details) based on student feedback. Skill performance was assessed at Millers’ level 3 (Show How) by an objective structured clinical examination (OSCE), and perceived competence at Dreyfus’ level 3 (Competence) using the retro-pre questionnaire.

Donald Kirkpatrick developed a four-level model of evaluation:

1) Reactions: measures how participants have reacted to the training.

2) Learning: measures what participants have learned from the training.

3) Behavior: measures whether what was learned is being applied on the job.

4) Results: measures whether the application of training is achieving results ^8– 10^.

The following instruments were used for assessment:

1. The retro-pre questionnaire for assessing learners’ self-reported changes. The retrospective post-then-pre design is a popular way to assess learners’ self-reported changes in knowledge, awareness, skills, confidence, attitudes or behaviors. It takes less time, is less intrusive and for self-reported change, avoids pre-test sensitivity and response shift bias that result from pre-test overestimation or underestimation ^11^.

2. A feedback questionnaire to assess the perception of teaching and learning sessions of S-PEST from students and faculty members.

3. The OSCE was used for the end of the posting assessment. Standardized patients (SP) were used in the OSCE. They were trained to follow students’ commands for various aspects of the physical examination. SP were healthy individuals from our house keeping department who consented to be SP. They were given prior briefing regarding appropriate mannerisms and how to respond to students’ commands during OSCEs. A faculty observer at each station used a checklist to rate each student’s performance.

The following components were developed by the station authors for each OSCE station:

- An instruction sheet for the examinee.
- A checklist for the assessment of the skill being examined at that station.
- A detailed patient profile for the standardized patient.
- A list of the equipment, instruments etc required at the station.

### VII. Data management and analysis

Data was analysed using SPSS version 18.0.

### VIII. Ethical considerations

The institutional Research Committee of KIST Medical College approved the project.

## Results

### A. Students’ self-reported changes in perceived skill levels using the retro-pre questionnaire

Forty-six skills, representing various systems in different departments during the training were included in the retro-pre questionnaire. Two to four skills from the ‘must know’ category from each subject/chapter were included in this questionnaire. Each skill was scored out of 4. Individual skill scores were added to get overall scores. A paired t-test was used to evaluate the difference in overall scores before and after the module. Scores were found to follow a normal distribution as confirmed by a Shapiro-Wilk test.

The average perceived skills score (the maximum score being 184) before the module was 15.9, which increased to 116.5 after the module. Students perceived that their level of skill improved after the module. The result from the paired t-test showed that the difference is highly statistically significant (mean 100.5 with SD+/- 23 and 95% confidence interval 95.45 – 105.59), which means that students did learn most of the skills after the Structured Physical Examination Skills Training (S-PEST) module and it did influence them.

Retro-pre questionnaire and scores Student self-evaluation of confidence in key skills using a retro-pre questionnaire before and after the introduction of the structures physical examination skills module at KIST Medical College, Nepal. The before scores are between 1-5: 1-being not aware, 2-not confident, 3-Somewhat confident, 4-very confident, 5-can do independently The after scores are between 1-4: 1-not confident, 2-somewhat confident, 3-very confident, and 4-can do independently

### B. Feedback of students regarding S-PEST

Around 75% of students filled in the feedback questionnaire which used a five point Likert scale. The questions were on the objectives of the session, facilitator/preceptor role, satisfaction with learning activities in each sub-session, overall rating of session and there were two open ended questions (“Suggestion/s for improvement” and “Any other comment/s”). Respondents gave scores out of 5, the higher the score, the higher the satisfaction.

**Figure 1.**
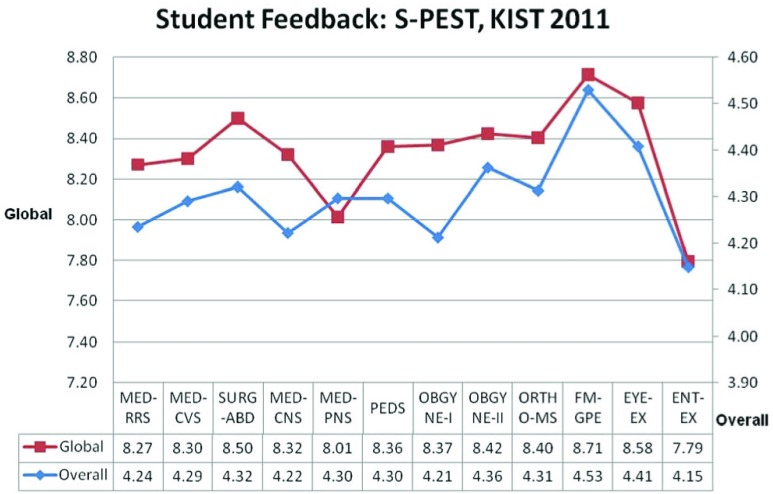
The graph shows the feedback of the students on Structured Physical Examination Skills Training (S-PEST) on skills imparted in various systems and delivered by different departments. Overall scores refer to the average student feedback scores (out of 5) in response to seven questions for each session. Global scores refer to the average student feedback scores (out of 10) for the importance of each session as a whole. Students agreed that they learned the skills of all the systems in various departments and they strongly agreed learning General Physical Examination skills in the Family Medicine department. Session abbreviations: MED-RRS – Medicine-Respiratory System; MED-CVS – Medicine-Cardiovascular System; SURG-ABD – Surgery-Abdomen; MED-CNS – Medicine-Central Nervous System; MED-PNS – Medicine-Peripheral Nervous; PEDS = Pediatrics; OBGYNE-I – Obstetrics; OBGYNE-II – Gynecology; ORTHO-MS – Orthopedics-Musculoskeletal System; FM-GPE – Family Medicine-General Physical Examination; EYE-EX – Eye Examination; ENT-EX – Ear Nose Throat Examination.

The average scores of the different questions and global scores (overall session ratings) were calculated. The average feedback score was found to be 4.30 (maximum score 5) and the overall global score was 8.34 (maximum score 10) (see Figure 1 for scores and average scores). The agreement between global (subjective) and overall items average (objective) scores were found to be 78% (Pearson’s Correlation).

Almost all respondents recognized the importance of each sub-session (Tutorial, Demonstration and Practice). The most frequent remark obtained from the open-response category question was to “provide more time for practice”.

SPEST questionnaires and student feedback scores for each section Student feedback for the introduction of structured physical examination skills at KIST Medical College, Nepal derived from an SPEST questionnaire for both students and faculty. Only student data is shown as the faculty SPEST results are mainly qualitative.
1st data file: Scores were provided out of 5 for questions i-vii (1=strongly disagree; 5=strongly agree), whilst the overall score for each section was given out of 10 (1 being poor and 10 being excellent).
2nd data file: Proportion of students for each score provided in response to the questions "I would rate the session........on a scale of 1 to 10 (1 being the least and 10 being the most important)”.
3rd data file: Proportions of students preferring either the tutorial, demonstration, practice or all three sub-sessions for each session.

### C. Feedback of faculty members on S-PEST

The feedback questions were on areas for improvement, how sessions could be improved, what could have been done differently and the faculty members’ perceptions of the development of reasoning and diagnostic skills early on in the students’ clinical years. Fifty-six feedback forms were received from faculty members. The frequency of each item scores on perception of teaching/learning sessions conducted as per protocol together with students’ clinical reasoning and diagnostic skills developed early in clinical years was calculated. None of the faculty members strongly disagreed, one (1.8%) disagreed, 2 (3.6%) remained neutral, 33 (58.9%) agreed and 20 (35.7%) strongly agreed.

The most frequent comments obtained from the open response category questions were:

I. Areas for improvement:

1) Students require more time for practice.

2) Decrease group size.

3) Increase number of patients available for teaching-learning.

II. How sessions could be improved:

1) More time required for demonstration on patients.

2) Models may be used for demonstration and practice.

III. What could be done differently:

1) Using videos of PE.

2) Demonstration on manikins.

3) Teaching on models.

### D. End of posting assessment using OSCE

**Figure 2.**
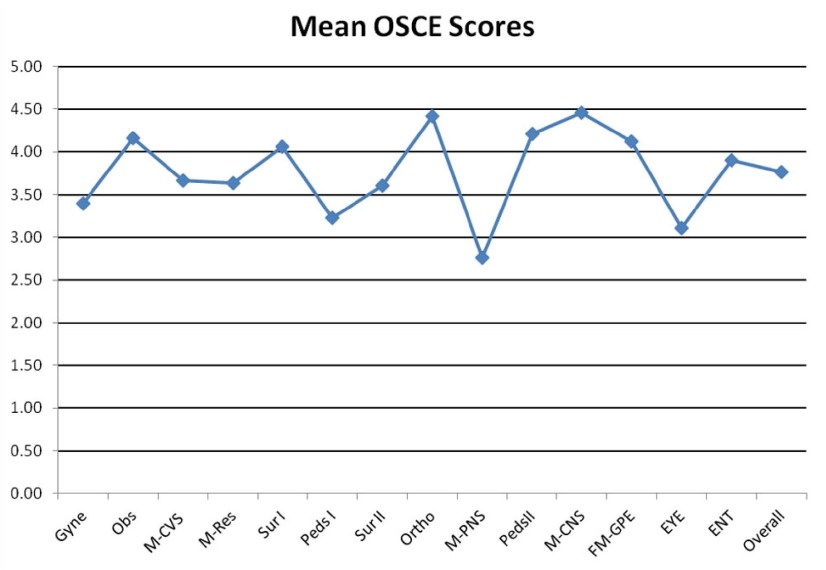
The mean total Objective Structure Clinical Examination OSCE score obtained by students for each session (out of 5). Session abbreviations: Gyne – Gynecology; Obs – Obstetrics; M-CVS – Medicine-Cardiovascular System; M-RES – Medicine-Respiratory System; Sur I – Surgery-Abdomen-liver; Peds I – Pediatrics I; Surgery II – Abdomen-kidney; Ortho – Orthopedics-Musculoskeletal System; M-PNS – Medicine-Peripheral Nervous System; Peds II – Pediatrics II; M-CNS – Medicine-Central Nervous System; FM-GPE – Family Medicine-General Physical Examination; EYE – Eye Examination; ENT – Ear Nose Throat Examination.

Out of 100 students, 98 attended the OSCE. There were 14 OSCE stations; each representing a different system (CVS, RSS, PNS, CNS, Abdomen I & II, Obstetrics, Gynecology, Pediatrics I & II, MS, General PE, Eye, and ENT). The mean total OSCE score obtained by students in each station was 3.77 with a standard deviation (SD+/-) of 0.33 (the maximum score was 5). Eighty percent of the students scored more than 70% (26 students scored more than 80%, 55 students between 70 and 80%, and 15 students between 60% and 70%). A graph of the OSCE scores is shown in Figure 2.

OSCE results Anonymized objective structured clinical examination (OSCE) student scores for each section at KIST Medical College, Nepal.

## Discussion

Clinical skills acquisition is a major focus of education for health professionals extending from undergraduate to postgraduate and continuing to professional education ^12^.

The current trend in medical education is to introduce clinical teaching early, within the first two years of the curriculum, to help students understand the relevance of the basic sciences to clinical practice and to provide instruction in basic clinical skills in a standardized fashion ^13^.

Following these trends, Tribhuvan University (TU), Kathmandu, included early clinical exposure (ECE) in the curriculum, revised in 2008. The clinical examination, a required course for second year students, concentrates on the teaching of physical examination skills in two departments (Medicine and Surgery) with no defined method of teaching/learning and assessment ^3^.

The importance of structured clinical education has long been recognized. It provides equal learning opportunities and a suitable environment for everyone to acquire clinical skills and competencies. Modules are especially suitable for outcome-based adult learning programs and maximizing adult learning ^14, 15^. With this purpose in mind, a teaching/learning module was developed in this project at the KIST Medical College Nepal KISTMC (affiliated to TU) for teaching basic physical examination skills to second year students as part of early clinical exposure. KISTMC involved 8 clinical science departments and made teaching, learning and assessment structured.

Teaching-learning was conducted in small groups as small group teaching and learning is considered effective in clinical settings for tutorials and demonstrations ^16^.

Physical examination skills are largely psychomotor skills. For teaching physical examination skills, Irby’s three stages of clinical teaching were applied (Preparation, Teaching and Reflection) ^5, 17^. Though faculty members and students reflected on experiences at the end of each session, these reflections could not be recorded ^5^.

All the faculty members involved in teaching received teacher-training before commencement of the course and were oriented about the implementation of the project ^18^.

Students were well informed about the project implementation but a limitation was that the students’ stage of competency could not be assessed at the beginning of the project (this is why the retro-pre questionnaire was used).

Skill acquisition was based on Millers’ Learning Pyramid at the ‘Show how level’ and Dreyfus’ competency model at the ‘Competent level’ (i.e. consciously competent) ^5, 6^.

Based on the method used for teaching clinical skills in the American College of Surgeon’s advanced trauma life support course, a tutorial, demonstration and practice (TDP) model was developed because of the limited time allocated for demonstration and practice session.

Feedback both from faculty members and students was taken on teaching and learning. All students were satisfied with the S-PEST. Almost all recognized the importance of each sub-session (tutorial, demonstration and practice). Students agreed that they learned the skills of all the systems but suggested more time to be provided for practice. Sir William Osler (1849–1919) gave emphasis to practice. He said:

“ *Observe, record, tabulate, communicate. Use your five senses… Learn to see, learn to hear, learn to feel, learn to smell, and know that by practice alone you can become expert*” ^19^.

Faculty members too were generally satisfied with the S-PEST. They commented that with the implementation of this module, students’ clinical reasoning and diagnostic skills seemed to develop early on in the students’ clinical years. Faculty members too felt that the students required more time for practice. They suggested that models, manikins and videos may be used for demonstration in addition to real and simulated patients.

Patsy Stark and F. Fortune had previously suggested that models may not be appropriate for teaching/learning skills but manikins and videos could be used instead ^20^. They suggested that dedicated and structured clinical skills training is the most important factor, whether it takes place in a skills centre, in the ward or in the community ^20^. A significant improvement in first-year medical student performance on the adult PE occurred after the use of a web-based instructional video at the University of Connecticut, School of Medicine, USA ^21^.

In this study, assessment of skills training effectiveness was done at level 1 (Reaction) as per Kirkpatrick’s model from students through feedback and skill performance done at level 3 (Show How) as per Miller’s pyramid model of demonstrated learning by OSCE and perceived competence at level 3 (competent) as per Dreyfus competency model of skill performance through the use of the retro-pre questionnaire ^6, 8– 10, 22^.

Analysis of our retro-pre model in line with the Dreyfus model of skill acquisition suggests that students did learn most of the skills following the implementation of the S-PEST Module. One limitation is that although the retro-pre model may reveal valuable information, it is not a substitute tool for an objective measure or a gold standard, but can be used where a large number of skills are to be assessed at one point in time. Don W. Scott *et al.* from the University of Chicago Pritzker School of Medicine used retro-pre modelling for assessing teaching skills and they recommended this method for assessment ^23^.

The OSCE is a proven valid and reliable, formative and summative tool for assessing the clinical skills learned by health sciences students ^24^. Standardized patients (healthy individuals trained to portray all the characteristics of an actual patient and to provide constructive feedback) were used in the OSCE. Students did well in the OSCE but one limitation was that only one skill from each system was assessed out of several skills taught in each system. Students and Faculty members also seemed to be satisfied with the OSCE process.

## Conclusion

Students that were introduced to S-PEST acquired basic PES used to examine medical, surgical, gynecological and obstetric, orthopedic, ENT, and eye adult patients, as well as pediatric patients. It is expected that S-PEST will enhance medical students' performance during their clinical years. In this study, the students did well in OSCE and both students and Faculty members were satisfied with the training and assessment.

## Limitations

The main limitations of this study were:

1. Limited time was allocated for each training session;

2. A pre-test for students’ stage of competency could not be done;

3. Both real and simulated patients were used for demo and practice;

4. Reflection on experiences at the end of each session could not be documented; and

5. In OSCE, only one skill from each system was assessed out of several skills taught.
