# Supplementary material for: Introduction of structured physical examination skills to second year undergraduate medical students
Source: F1000Res. 2013 Jan 16;2:16. [Version 1] doi: 10.12688/f1000research.2-16.v1 (PMC3901509; doi:10.12688/f1000research.2-16.v1)
Supplement: Retro-pre questionnaire and scores — Student self-evaluation of confidence in key skills using a retro-pre questionnaire before and after the introduction of the structures physical examination skills module at KIST Medical College, Nepal. The before scores are between 1-5: 1-being not aware, 2-not confident, 3-Somewhat confident, 4-very confident, 5-can do independently The after scores are between 1-4: 1-not confident, 2-somewhat confident, 3-very confident, and 4-can do independently [file f1000research-2-1206-s0002.tgz › A_Retrospective_Pre_questionnaire_Influence_of_Teaching_Structured_Physical_Examination_Skills.pdf]

Influence of Teaching Structured Physical Examination Skills Training- A Retrospective Pre questionnaire

		Before Training					After Training			
	**Skills**	**Not aware**	**Not confident**	**Somewhat confident**	**Very Confident**	**Will be able to do independently in future**	**Not confident**	**Somewhat confident**	**Very Confident**	**Will be able to do independently in future**
1	Measuring body temperature									
2	Measuring Respiratory Rate									
3	Recording Pulse									
4	Measuring Blood Pressure									
5	Detecting Pedal Edema									
6	Detecting Cyanosis									
7	Detecting Clubbing									
8	Palpating cervical Lymph Nodes									
9	Demonstrating range of motion at elbow Joint									
10	Demonstrating range of motion at Hip Joint									
11	Palpating Peripheral Pulses									
12	Assess expansion of the chest									
13	Determining Position of Trachea or palpation for Trachea									
14	Percussion of Anterior Chest									
15	Auscultation of Posterior Chest									
16	Eliciting Vocal Resonance on anterior chest									
17	Measuring Jugular Venous Pressure									
18	Localizing of Apex Beat									
19	Auscultation of Precordium									
20	Use of otoscope in examining ear									
21	Use of nasal speculum in examining ear									
22	Use of head mirror with light in examining ear									
23	Measurement of Visual Acuity									
24	Measurement of Visual Fields by confrontation method									

		Before Training					After Training			
	**Skills**	**Not aware**	**Not confident**	**Somewhat confident**	**Very Confident**	**Will be able to do independently in future**	**Not confident**	**Somewhat confident**	**Very Confident**	**Will be able to do independently in future**
25	Examination of unioccular eye movement (Muscles)									
26	Examination of Pupillary Light Reaction									
27	Palpation of Liver									
28	Palpation of Kidneys									
29	Palpation of Spleen									
30	Eliciting of Shifting Dullness									
31	Measurement of Liver Span									
32	Examination of Facial Nerve									
33	Examination of Accessory nerve									
34	Eliciting Tone of Muscles in Upper Limbs									
35	Eliciting Reflexes in Lower Limbs									
36	Eliciting Planter Response									
37	Eliciting Position Sense in Lower Limbs									
38.	Assessing orientation to time, place and person									
39	Measurement of Height in Children									
40	Measurement of Mid arm Circumference in Children									
41	Demonstration dehydration in infants									
42	Counting Respiratory rate in children under five years of age									
43	Demonstration of pallor in children									
44	Demonstration of jaundice in children									
45	Measurement of Height of Fundus									
46	Eliciting obstetrical grips or Leopold's maneuver									
