# Supplementary material for: Introduction of structured physical examination skills to second year undergraduate medical students
Source: F1000Res. 2013 Jan 16;2:16. [Version 1] doi: 10.12688/f1000research.2-16.v1 (PMC3901509; doi:10.12688/f1000research.2-16.v1)
Supplement: SPEST questionnaires and student feedback scores for each section — Student feedback for the introduction of structured physical examination skills at KIST Medical College, Nepal derived from an SPEST questionnaire for both students and faculty. Only student data is shown as the faculty SPEST results are mainly qualitative. 1st data file: Scores were provided out of 5 for questions i-vii (1=strongly disagree; 5=strongly agree), whilst the overall score for each section was given out of 10 (1 being poor and 10 being excellent). 2nd data file: Proportion of students for each score provided in response to the questions "I would rate the session........on a scale of 1 to 10 (1 being the least and 10 being the most important)”. 3rd data file: Proportions of students preferring either the tutorial, demonstration, practice or all three sub-sessions for each session. [file f1000research-2-1206-s0001.tgz › Questionnaire__for_Feedback_of_Faculty_on_Structured__Physical_Examination_Skills__Training.pdf]

# “Structured Physical Examination Skills Training”

## Feedback on the session from faculty

Department: ……………………….   System: ………………………………..

Session: I / II / III / IV   Group: A / B / C / D / E / F / G   Subgroup: 1 or 2

1. Which sub-session/s went well: i) Tutorial ii) Demonstration iii) Practice iv) All
   (Encircle you believe)

2. Areas of improvement ………………………………………………………………………………………………………………..

3. How it/these could be improved ………………………………………………………………………………………………………

4. What could be done differently ……………………………………………………………………………………………………….

5. Do you think these teaching/learning sessions conducted as per protocol and with these teaching/learning sessions Students’ clinical reasoning and diagnostic skills developed early in clinical years 1= strongly disagree, 2= disagree, 3= neutral, 4= agree, 5= strongly agree (encircle one)

Name of faculty (Optional) ………………………………………………………………………………………………………
