# Supplementary material for: Introduction of structured physical examination skills to second year undergraduate medical students
Source: F1000Res. 2013 Jan 16;2:16. [Version 1] doi: 10.12688/f1000research.2-16.v1 (PMC3901509; doi:10.12688/f1000research.2-16.v1)
Supplement: Peer review history

# Peer review: Referee response for version 1

**Published:** 2013-06-07

**Authors:** Bruce Fisher

**Affiliations:** 1 Division of General Internal Medicine, University of Alberta, Edmonton, Alberta, Canada

**Title and Abstract:** The title is acceptable and clear.

**Article content:** The authors are to be applauded for their thoroughness and attention to accepted pedagogical models when designing and setting up their curriculum. However, there is little description of the actual content and emphasis of the curricular components, and the methods by which key knowledge and skills were actually assessed in the OSCEs. It is not clear if the OSCEs had been validated, or whether there was any comparison group for analysis of effect. There was no information to determine if the OSCE was used in any novel way. 

**Data:** The methods used to introduce the curriculum were presented in some detail, but as stated above there were significant areas not described. The figures were unhelpful and the data could be better portrayed in table form. 

**Conclusions: **The conclusions were substantiated by the data presented, but without information about any comparison or control group, it is difficult to gauge the impact of the curricular intervention (over that of secular trend etc).  It is also not clear what these conclusions add to the existing literature.

I have read this submission. I believe that I have an appropriate level of expertise to confirm that it is of an acceptable scientific standard, however I have significant reservations, as outlined above.

# Response

**Published:** 2013-06-08

**Authors:** Rano Mal Piryani

**Affiliations:** KIST Medical College Lalitpur Kathmandu Valley Nepal, Nepal

Dear Bruce Fisher 

Thanks for reviewing article and providing suggestions for update. 

**Article content:** Due to word limitation criteria, every detail of content cannot be included in the article; however, adequate information is provided in the introduction, methods and result section. Curricular components are mentioned briefly in the introduction. Contents are described briefly in method section under development of module, orientation of faculty members, approach to teaching, approach to learning, teaching/learning strategies and assessment section. OSCE was validated, the details of which are mentioned in the comments in response to Dr. Deborah Korenstein (See comments in response to referee 2).

 

OSCE was conducted in a novel way. The summary of practical arrangements for OSCE at KIST Medical College is as follows:

 

**1. Prior to the OSCE session.**

- Suitable venue was selected.
- Assessors/examiners were identified.
- Standardized Patients were selected.
- Running order of the stations in circuit was developed.
- List of equipments/instruments required was prepared.
- Tasks, checklists, feedback questionnaires etc printed 

 

**2. The day before the OSCE session**

- Inspection of OSCE stations done
- Stations were clearly labeled
- Condition of required equipments/instruments was checked
- A pack of the documents for the OSCE examiners, students and patients were made available
- Signs were displayed at proper place

**3. On the day of the OSCE session**

- Reliable stop watch and loud manual bell were used
- Support staffs were placed to direct the candidates, examiners, and SPs
- Assessors explained SPs about their role once again
- Students were briefed
- Supervisors observed the session
- Feedback was taken from students, assessors and SPs 

 

**4. At the end of the OSCE session**

- Feedback questionnaires were collected
- Checklists were collected
- Token money paid to SPs
- Contributions of everyone was appreciated 

 

**5. After the OSCE session**

- Score was compiled and result declared
- Feedback questionnaires data compiled

 

**Data:** All the available data is included in data files of the article.

**Conclusions:** The conclusions are sensible, reasonable and rational.

Rano Mal Piryani 

Principle Author

# Peer review: Referee response for version 1

**Published:** 2013-05-29

**Authors:** Deborah Korenstein

**Affiliations:** 1 Division of General Medicine, Mount Sinai School of Medicine, New York, NY, USA

**Title and Abstract:** The title is OK, though I would describe it as a structured physical exam skills *educational program* (italicized words added by me). The abstract focuses largely on the theoretical models underpinning the approaches to evaluation. I think it would be clearer to focus concretely on concisely describing the evaluation performed. The theoretical rationale for that approach can be described in the paper itself.

**Article content:** Overall, the methods and design are adequately explained, though there are a few important exceptions. The authors go into great detail about the theoretical models underpinning their approach to evaluation. This is fine but is not really critical. The critical components of both the survey and the OSCE are whether these are validated instruments. I assume the authors created both for this project, but it is important for them to state that these tools have not been previously validated (unless of course they have). In addition, I did not find the Figures particularly helpful. They might be more clearly displayed in table format than as graphs.

**Conclusions:** The conclusions are reasonable, though I would add the caveat that because the measurement instruments were not validated it is difficult to be certain of their reliability. Also, the authors should emphasize that the meaning of the OSCE scores in particular is not clear since there is no comparison group and the OSCE has not been validated.

**Data:** The data is limited, but the curriculum design is presented in adequate detail.

I have read this submission. I believe that I have an appropriate level of expertise to confirm that it is of an acceptable scientific standard, however I have significant reservations, as outlined above.

# Response

**Published:** 2013-06-08

**Authors:** Rano Mal Piryani

**Affiliations:** KIST Medical College Lalitpur Kathmandu Valley Nepal, Nepal

Dear Deborah Korenstein, 

Thanks for reviewing article and providing suggestions for update. 

**Title and Abstract: ** As suggested evaluation performed is added in abstract concisely.  

**Article content: ** Both the survey ****and the OSCE were validated. Details of validation could not be included in article because of word limits. Validation of OSCE process was done in steps mentioned below. 

**Step I:**  OSCE academic team was formed. Preliminary workshop on OSCE was arranged in May 2010 for 3 hours for drafting OSCE stations (development of all three components of OSCE station--tasks, checklists and instruction/direction to simulated patients) utilizing standard book of clinical methods and evidence based literature. For drafting of tasks and checklists The Hutchison's Clinical Methods 22nd edition published in 2007 was chosen as standard book. Faculty members involved in teaching physical examination skills were instructed to draft two OSCE stations for each system within 8 weeks time. 

**Step II:** Second workshop of OSCE academic team for two half days was organized in July 2010 to assess and finalize all three components of OSCE stations, develop instructions for examiner/assessor and scoring (marking) criteria. Editing of all three components of OSCE stations was done by experienced senior faculty members. 

**Step III:** All the documents developed in second workshop printed in first week of August for conducting OSCE session in the end of August 2010 for first batch of undergraduate students **.**

**Step IV:** OSCE session conducted in the last week of August 2010 and the written feedback obtained from the examiners/assessors, faculty involved in teaching physical examination skills and students. This session was considered as piloting for OSCE session to be conducted for batch II in 2011 for proposed project. 

**Step V:** Feedback taken from the examiners/assessors, faculty involved in teaching physical examination skills and students compiled, analyzed and shared with OSCE academic team in a meeting arranged in September 2010. Also score obtained on each station and total score by students shared in the same meeting. The team fully satisfied with the entire process and made some recommendations for conducting session for batch II in 2011 for proposed project.   

**Step VI** ****OSCE academic team developed in 2010, reactivated in May 2011. Faculty members involved in teaching were assigned to review all three components of OSCE stations i.e. Tasks (stem), checklists and instruction/direction (training information) to simulated patients used in 2010 for assessment of first batch technically and as well as in the light of recommendation of OSCE academic team for conducting next session. OSCE Academic Team in depth reviewed all three components of OSCE stations in July 2011 in meeting cum workshop for two half days, edited & finalized. The documents printed in August 2011 and OSCE session held on September 27, 2011 for second batch (for project). 

**Conclusions:** Validated tools were used for assessment **. **Mean of score obtained by students (98 students) on each OSCE station was calculated. Then overall mean was calculated. This data is included in article. 

**Data:** All the available data is included in data files of the article.

Rano Mal Piryani

Principle author

# Peer review: Referee response for version 1

**Published:** 2013-04-08

**Authors:** Ramesh K Adhikari

**Affiliations:** 1 Department of Child Health, Institute of Medicine, Tribhuvan University, Kathmandu, Nepal

The title reads more like a description of an activity, suggesting that it reflects the article which reports on effectiveness of the TDP model in improving PES.

In regards to the content, I would suggest that the authors explain the 'retro- pre-questionnaire' in some detail. When did they administer this questionnaire and if there is any relationship between the responses to the questionnaire and OSCE? 

The conclusions are sensible and balanced.

The data are satisfactory to justify the conclusions.

I have read this submission. I believe that I have an appropriate level of expertise to confirm that it is of an acceptable scientific standard.

# Response

**Published:** 2013-06-08

**Authors:** Rano Mal Piryani

**Affiliations:** KIST Medical College Lalitpur Kathmandu Valley Nepal, Nepal

Dear Ramesh Adhikari   

Thanks for reviewing article and providing suggestions for update. The title of the article was finalized after consultation with all authors. A pre test was not done, so 'retro- pre-questionnaire' was used for assessing learners, i.e. self reported changes in acquiring physical examination skills. This was done at the end of project, before OSCE session. This is mentioned in the article. No correlation was done between response of retro-pre and OSCE score.

Rano Mal Piryani

Principle Author
